# Physical, Mechanical, and Biological Properties of Fibrin Scaffolds for Cartilage Repair

**DOI:** 10.3390/ijms23179879

**Published:** 2022-08-30

**Authors:** Juan Antonio Rojas-Murillo, Mario A. Simental-Mendía, Nidia K. Moncada-Saucedo, Paulina Delgado-Gonzalez, José Francisco Islas, Jorge A. Roacho-Pérez, Elsa N. Garza-Treviño

**Affiliations:** 1 Laboratorio de Terapia Celular, Departamento de Bioquímica y Medicina Molecular, Facultad de Medicina, Universidad Autónoma de Nuevo León, Monterrey 64460, NL, Mexico; 2 Servicio de Ortopedia y Traumatología, Hospital Universitario “Dr. José Eleuterio González”, Universidad Autónoma de Nuevo León, Monterrey 64460, NL, Mexico; 3 Departamento de Hematología, Hospital Universitario “Dr. José Eleuterio González”, Universidad Autónoma de Nuevo León, Monterrey 64460, NL, Mexico

**Keywords:** fibrin, articular cartilage, scaffold, biomaterials, cartilage engineering

## Abstract

Articular cartilage is a highly organized tissue that provides remarkable load-bearing and low friction properties, allowing for smooth movement of diarthrodial joints; however, due to the avascular, aneural, and non-lymphatic characteristics of cartilage, joint cartilage has self-regeneration and repair limitations. Cartilage tissue engineering is a promising alternative for chondral defect repair. It proposes models that mimic natural tissue structure through the use of cells, scaffolds, and signaling factors to repair, replace, maintain, or improve the specific function of the tissue. In chondral tissue engineering, fibrin is a biocompatible biomaterial suitable for cell growth and differentiation with adequate properties to regenerate damaged cartilage. Additionally, its mechanical, biological, and physical properties can be enhanced by combining it with other materials or biological components. This review addresses the biological, physical, and mechanical properties of fibrin as a biomaterial for cartilage tissue engineering and as an element to enhance the regeneration or repair of chondral lesions.

## 1. Introduction

Articular cartilage is a highly organized tissue that provides remarkable load-bearing and low friction properties, allowing for smooth movement of diarthrodial joints [1]. Joint cartilage contains sparsely distributed chondrocytes embedded within the extracellular matrix (ECM). The ECM is mainly comprised of water, type II collagen, and glycosaminoglycans that provide the tissue with sufficient mechanical properties for several biological functions, such as load-bearing and low friction [2,3]. Due to the avascular, aneural, and non-lymphatic characteristics of cartilage, joint cartilage has self-regeneration and repair limitations [2]. When the cartilage gets damaged, if the diameter of the injury is greater than 4 mm, spontaneous self-repair capacity becomes limited [4]. Moreover, focal cartilage lesions predispose to developing early-onset osteoarthritis, which may lead to long rehabilitation periods and loss of function.

Treatment for cartilage injuries focuses on relieving pain [5]. It may include lifestyle changes, oral anti-inflammatories [6], physical therapy [7], intra-articular injections of hyaluronic acid or steroids [8], bisphosphonates [9], and even surgical interventions such as joint replacement [7]. Cell therapy is currently being proposed as an alternative strategy. In focal cartilage lesions, autologous chondrocyte implantation and mesenchymal stem cells (MSC) seeded onto scaffolds have been used to restore these defects with good results [10]. Other cell lines that have been used to repair focal cartilage lesions by implantation into the lesion site are embryonic stem cells and induced pluripotent stem cells (iPSCs) [11]; however, even though cell therapy has limited therapeutic activity, one of its main advantages is the short-term reduction in clinical symptoms. Thus, medicine has turned to a tissue engineering approach to prolong the therapeutic effect of cell therapy.

The successful use of tissue engineering techniques to form engineered cartilage is based on a combination of three critical elements: a cellular component, a bioactive component (such as growth factors), and a biocompatible and mechanically stable carrier vehicle/matrix scaffold [12]. The cellular component consists of healthy, viable cells that are accessible, manipulable, and nonimmunogenic. The bioactive component should promote the differentiation and maturation of the cellular component. The carrier has a dual function, acting as both a delivery vehicle and a scaffold [13]. In addition, the carrier should provide sufficient mechanical support to withstand in vivo forces [14] and must be degraded by the cells, giving way to its replacement or contributing to the formation of new tissue [15,16,17].

The basic types of biomaterials used In tissue engineering can be classified in relation to their origin as synthetic (usually chemical-nature materials) and natural (derived from biomolecules, tissues, or living organisms) [18] depending on its structural patterns as a polymer (composed of many repeated subunits) and a composite (a combination of two different biomaterials, a polymer, and a filler) [19]. Natural polymers such as collagen, silk fibroin, and fibrin are some of the most common, used as scaffolds for cartilage engineering. Fibrin is one of the most promising natural biomaterials for articular cartilage repair [20]. Fibrin polymers and composites have been used to induce regeneration as a vehicle for bioactive molecules to promote injury healing and delivery carriers for multiple cell lines [21]. Moreover, they are not expensive and are easy to obtain from whole blood.

In this review, we focused on analyzing the physical, mechanical, and biological properties of fibrin scaffolds, one of the most promising biomaterials in cartilage engineering due to its advantages over other common biomaterials used in the field. We pointed out the advantages and disadvantages of the utilization as a scaffold on its own or in combination with other biomaterials and cells to maximize their properties such as biocompatibility, biodegradation, mechanical resistance, and improved ability to repair chondral tissue.

## 2. Fibrin: Structure and Molecular Interactions

Fibrin is a native biopolymer derived from fibrinogen [22,23]. Fibrinogen is a blood component that plays an important role in hemostatic function. It is also related to cellular processes such as proliferation, differentiation, adhesion, migration, healing, inflammation, and angiogenesis [24]. Fibrinogen is described as a long glycoprotein (340 Kda) made up of a dimer of three disulfide-linked polypeptide chains called Aα (66,500 Da), Bβ (52,000 Da), and γ (46,500 Da) [22,25]. Fibrinogen consists of two globular D regions and one central globular E region, each with a part of α-helical coiled-coils.

**Figure 1:**
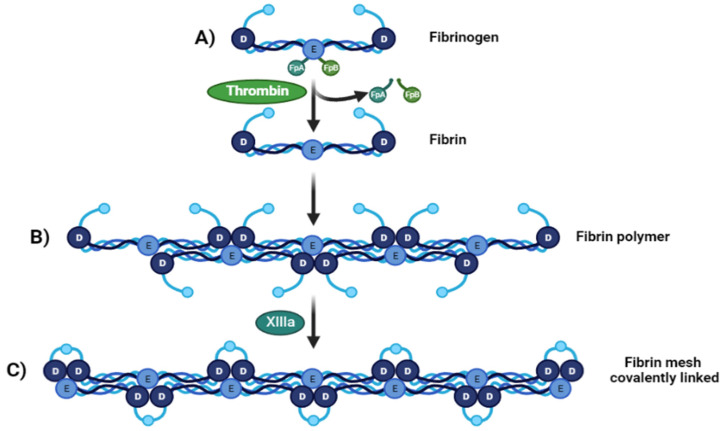
From fibrinogen to fibrin Mesh. (**A**) Fibrinogen D:E:D regions interact with thrombin-realizing fibrinopeptides (FpA and FpB) (**B**). Soluble fibrin is then activated by Factor XIIIa, permitting sulfide bonding to crosslink among fibrin, converting it to a (**C**) crosslinked fibrin polymer.

Fibrinogen is transformed into fibrin monomers during blood clotting due to thrombin [23], which cleaves fibrinopeptide A (FpA) and fibrinopeptide B (FpB) from the N-terminal sites of the Aα and Bβ chains of fibrinogen, respectively Figure 1A. At this point the fibrin fibers made of two fibrin nano peptides half-staggered with a crystalline-like structure, can reach a size of 100 nm, After FpAs cleavage each α chains have a new sequence on the N-terminal (Gly-Pro-Arg) called knobs “A” [26], then these fibers can come together and form a crosslinked mesh. This initiates fibrin assembly by exposing a polymerization site called EA. Each EA site combines with a constitutive complementary-binding pocket in the D domain (Da) to form the initial EA:Da association by forming intermolecular ε-((γ-glutamyl) lysine bonds, causing double-stranded twisting fibrils by aligning in a staggered overlapping end-to-middle domain arrangement [27]. The g-g crosslinks form reciprocally between glutamine 398 or 399 and lysine 406, and other flexible bonds form such as a–a crosslinks Gln-221, -237, -328, -366 and Lys-418, -448, -508, -539, -556, -580, and -601, all conferring particular mechanical and elastic properties [28] (Figure 1B). The C-terminal region of each fibrinogen or fibrin c-chain contains one crosslinking site at factor XIII or XIIIa. These give fibrin structural integrity and stabilize the clot against proteolytic and mechanical insults because of isopeptide bond formation, passing from a soluble state to an insoluble one crosslinked by ε-(γ-glutamyl)-lysine stable covalent bonds [25] (Figure 1C).

All the molecular and structural properties mentioned above allow crosslinking of the fibrin with different biomaterials, enhancing fibrin mechanical and elastic properties, and generating new biomaterials and scaffolds that resemble the physical properties of articular cartilage.

## 3. Mechanical and Physical Properties of the Fibrin Scaffolds

Due to the mechanical resistance, elastic, and mesh-like nature, fibrin has been used as a sealant for surgical procedures and recently as hydrogel scaffold for cartilage engineering. Generally, fibrin scaffolds can be manufactured in three forms: fibrin glues, fibrin hydrogels, and fibrin microbeads [13]. The fibrin glues are obtained from plasma cryoprecipitate (which contains fibrinogen, fibronectin, and XIIIa factor). The cryoprecipitate is mixed with thrombin and calcium to obtain a fibrin polymer, which can be used as a patch (2D) or as a 3D scaffold [29]. Fibrin hydrogels are made from purified fibrinogen, thrombin, and calcium salt. The main difference between fibrin glues and hydrogels is the presence of coagulable proteins in the fibrin glue [30]. On the other hand, fibrin microbeads are obtained from fragmented plasma and thrombin; however, polymerization takes place in an emulsifier at 75 °C where the fibrinogen gets denaturalized and the XVIIIa factor crosslinks the fibrin into a more stable and dense form [13]. In all the fibrin scaffolds, the mechanical strength will depend on the amount of fibrin and thrombin [31]; however, when it comes to fibrin composites, the fibrin becomes a filler and a functional part, and the mechanical strength will depend on the other component or phase of the biomaterial.

To increase their mechanical strength, the fibrin scaffolds have been combined with different biomaterials, such as poly lactic-co-glycolic acid (PLGA) [32,33,34], hyaluronic acid (HA) [35], chitosan–alginate [36], polycaprolactone (PCL) [37], and although the results have been promising, increasing approximately 60 times the fibrin mechanical strength, in some cases, they match articular cartilage (0.24–0.85 Mpa [38]); however, it has not yet been possible to develop a scaffold that can match all the properties of cartilage.

For example, an increase in the amount of fibrin leads to greater mechanical strength, but the pore size decreases (Table 1). Pore size must be appropriate for some cell types; for example, 150–250 µm are desirable for articular chondrocytes [39] and 200–300 µm for MSC [40] to promote cell proliferation and the preservation of chondrogenic differentiation into the scaffold, ensuring the diffusion of oxygen, nutrients, and products of metabolism.

Fibrin combinations with some biomaterials achieve a suitable pore size, but due to the chemical nature of the biomaterial [41], there is a lack of growth factors that can improve or maintain chondrogenesis, so these must be provided to the scaffold formulation [42]. Other combinations have achieved an accurate pore size and mechanical strength, but with a decrease in scaffold elasticity, as shown in Table 1.

Creating a three-dimensional scaffold does not guarantee the creation of high-quality new cartilage tissue on its own. Instead, some help is needed to improve or induce the generation of new tissue. There are also numerous molecular interactions and conditions that contribute to the mechanical resistance of the scaffold. Usually, the scaffold is seeded with a cellular component, and this can improve the mechanical properties through ECM production [43]. Depending on the approach, the cell component in the scaffold should replace part of the scaffold for new tissue. At this point, the ECM can add mechanical support, elasticity, and stiffness [44]. That is why all the properties should be tested with and without cells to know the limitations of scaffolds.

With the creation of different scaffolds for cartilage repair, it is critical to compare their properties to choose a therapeutic approach. However, it has been difficult to compare the properties of the scaffolds due to inconsistency in the number and type of tests performed to prove their functionality. Ideally, a minimum number of tests should be set to evaluate their functionality and compare with others.

**Table 1:** Physical and mechanical properties of composed scaffold with fibrin.

Scaffold	Fibrin/Fibrinogen Content (mg/mL)		Pore Size (µm)	Mechanical Strength (Mpa)	Longitudinal Elasticity (Youngs Modulus)		Reference
		Other Component Content			Elastic Modulus (kPa)	Elongation at Break (%)	
Fibrin glue (Tiseel)	67–106	-	-	≈0.0029	15	-	[45]
Fibrin glue (EVICEL)	55–85	-	-	0.0135	38	-	[45]
Fibrin hydrogel	5	-	9.7 ± 7.1	0.0034	-	-	[31]
Fibrin hydrogel	12.5	-	8.1 ± 5.3	0.0054	-	-	[31]
Fibrin hydrogel	25	-	6.4 ± 3.4	0.0109	-	-	[31]
Fibrin hydrogel	50	-	-	≈0.01	20	-	[46]
Hydrogel: Fibrin-PAAm	50	44.46% PAAm	-	≈0.052	120	≈55	[46]
Composite: Fibrin-PAAm-PCL		44.46% PAAm PCL as a core	-	≈0.16	150	≈22	[46]
Composite: Fibrin-collagen sponge	110	-	≈110	≈12	-	-	[47]
Composite: Fibrin-genipin crosslinked DCM-PVA	-	Genipin = 0.04 g/g DMC-PVA 70:30	22–95	-	14.7 ± 2.7	62.39 ± 6.56	[16]
Htdrogel: Fibrin-PLC-ECM	-	PCL = 28% ECM = 2%, 5%, and 10%	250–400	0.13–0.20	-	-	[37]
Hydrogel: Fibrin-PLC-ECM (salt leached)	-	PCL = 28% ECM = 2%, 5%, and 10%	<400	0.02–0.05	-	-	[37]
Advanced platelet-rich fibrin glue	-	-	-	0.17	≈70	≈25	[48]
Platelet.poor plasma-derived fibrin glue	-	-	-	0.13	≈70	≈15	[48]

poly-ε-caprolactone (PCL); poly-vinyl alcohol (PVA); devitalized cartilage matrix (DCM); extracellular matrix (ECM).

## 4. Biodegradation

One advantage of fibrin scaffolds is that they present a controllable degradation rate to match host tissue regeneration [49]. The fibrinolytic system is responsible for the natural degradation of fibrin, which is the insoluble form of fibrinogen. This degradation depends on the fibrinolytic activity of plasmin which produces a series of smaller fragments generally called fibrin degradation products [50]. Due to the relatively rapid degradation of fibrin and its intrinsic instability, its use as a scaffold could be problematic in cases where certain stability and resistance are required from the biomaterial, which is the case of articular cartilage repair. Fibrin shows desirable scaffolding properties over other polymers. Table 2 summarizes the properties of natural, synthetic, and polysaccharide polymers.

Fibrin scaffold resistance to degradation can be obtained by crosslinking the fibers or by adding fibrinolysis inhibitors [51]. Some molecules have been used for this purpose, including aprotinin, tranexamic acid, or aminocaproic acid [52]. Such modifications to the fibrin matrix do not interfere with the properties of the biomaterial. They preserve the characteristics that favor cell growth, adhesion, proliferation, and ECM formation [53].

Regarding the decellularization of tissues or scaffolds before proceeding to their clinical application, it is very important to apply a rigorous in vitro and in vivo assay to validate the host response to these scaffolds.

**Table 2:** Characteristics of some natural, synthetic, and polysaccharide polymers used in biomedical applications.

Polymer Type	Material	Properties			Advantages	Disadvantages	Ref.
		Toxicity	Biocompatibility	Immunogenicity			
Natural	Fibrin	Not reported	High	Non-immunogenicity	Properties of cell adhesive/binding	Quick rate of degradation; Poor biomechanical strength	[42,54]
	Collagen	Low	High	Low	Favorable for cell adhesion, proliferation, and ECM secretion	Physical and chemical variable properties Variable degradation	[54,55]
	Silk fibroin	Non-toxicity	High	Prolongated presence of silk may induce degradation that may prompt the immune response	Support for cell adhesion, proliferation, and vascularization	Moderately degradable	[54,56]
	Gelatin	Low toxicity	High	Non immunogenicity	Better infiltration, adhesion, spreading, and proliferation of cells	Low stability in physiological conditions	[54,57]
	Chitosan	Non-toxicity	Hemostatic potential	Low immunogenicity	Promotes adhesion, accelerates repair, and prevents formation of scar tissue	Poor mechanical strength and stability Low solubility Quick rate degradation in vivo	[54,58]
	Alginate	Non-toxicity	High	Non-immunogenic	Mimicking function of the extracellular matrix	Low adhesion, poor mechanical characteristics	[59]
	Hyaluronic acid	Non-toxicity	High	Non-immunogenic	Supporting migration of mesenchymal stem cells and epithelial cells Fill irregular defects	Poor biomechanical strength Low biodegradability in the crystalline phase	[60]
Synthetic	Polylactic acid (PLA)	Non-toxicity	High	Non-immunogenicity	High stress resistance High Young’s modulus Possibility of synthesizing in different forms	His depolymerization require excessive heating Local acidosis caused by biodegradation products	[54,61,62]
	Poly(ƹ-caprolactone) (PCL)	Non-toxicity	High	Low immunogenicity	Good mechanical properties Controls cell proliferation and angiogenesis	Low bioactivity	[54,63]
	Polyvinyl alcohol (PVA)	Non-toxicity	High	Low immunogenicity	Higher elasticity Similar tensile strength to human articular cartilage	Lack of cell-adhesive property.	[54,64]
	Poly(ethylene glycol) (PEG)	Non-toxicity	High	Non-immunogenicity	Elastic Bioadhesive	Creates insoluble networks	[55,65]

## 5. Biocompatibility

The generation of biocompatible and biomimetic tissue-like biomaterials is crucial to ensure the success of engineered substitutes for tissue repair. Biosafety and biocompatibility analysis using a comprehensive approach including histological, hematological, biochemical, and imaging approaches support the clinical uses of biomaterial implantations.

In the analysis of the biocompatibility of the fibrin scaffolds or any other biomaterial, many techniques have been developed to ensure compatibility between the patient and the biomaterial. For example, in vivo assays consist of scaffold implantation in an animal to evaluate tissue proliferation and repair after animal euthanasia. Some damage factors that can be evaluated are redness, swelling, bleeding, and exudation around the incision. An implant biopsy can be performed and analyzed by histological techniques. Histological staining can evaluate cell adhesion and proliferation inside the scaffold, as well as the presence of lymphocytes and phagocytes in the incision area, which are indicators of inflammation [4]. Some fibrin scaffolds [66,67] or fibrin mixed with other biomaterial are compatible in animal models; for example, scaffolds of fibrin/PLC (Wang et al., 2020), fibrin/polyurethane [68], fibrin/agarose [4] (Table 3). There are other parameters in addition to cell proliferation and immune cells infiltration that may help to evaluate the biocompatibility of fibrin scaffolds such as absorption, vascularization, hemocompatibility, or surface wettability.

Material reabsorption is dependent on variables like particle size and porosity; in the case of biphasic compounds, the proportion of their components. The porosity of this material allows vascularization, fluid diffusion, cell migration, and its gradual resorption by chondrocytes, MSC, or another type. An ideal particle diameter between 200 and 350 μm is recommended [74], with a porosity between 10 to 100 μm, which may impede liquid diffusion and material absorption in the defect produced. The resorption rate is also influenced by particle size. For example, fibrin gels have a fibrous structure with pore diameters on the order of 1 µm. However, fibrin/HA-MA hydrogel formulations (4 mg/mL and 6 mg/mL) [15] and fibrin-agarose tissue-like hydrogels produced a more sheet-like morphology with pore diameters in the 10–100 µm range. Crosslinking reinforcement of the fibrin scaffold with HA-MA improved the compressive modulus of fibrin/HA-MA hydrogel and increased its chondrogenic potential in terms of biosafety and biocompatibility for joint cartilage repair.

Hemocompatibility is a major criterion that limits the clinical applicability of biomaterials. It is important to assess the biomaterial in close contact with blood o derivates which include plasma, erythrocytes, leukocytes, and platelets [75]. It has been reported that PCL/fibrin (0:100), PCL/fibrin (10:90), PCL/fibrin (20:80), and PCL/fibrin (30:70) scaffolds were not destructive to erythrocytes (hemolysis rate < 5%) [76], which was lower than the standard recommended value established by the International Organization for Standardization (ISO) [66]. Therefore, each time a formulation or scaffold is made of a polymer, whether natural or synthetic, modified or not, these parameters must be assessed before their potential clinical use. The good surface wettability of biomaterials should also be considered as a factor that positively influences the cellular response [77].

## 6. Cells on Fibrin Scaffolds

The cell component in the scaffold becomes one of the most important parts because it can improve and accelerate the regeneration process. Additionally, biomaterials cannot only improve the biocompatibility of the scaffold, but also enhance cell culture on the scaffold [78]. Cell–fibrinogen interactions are complex and mediated by the interaction of cell binding receptors (integrin) and surface adhesion sites (ligands) [79]. During wound healing, fibrin clots interact with ECM proteins such as fibronectin and vitronectin [30], creating a network or provisional scaffold binding endothelial cells, leukocytes, platelets and plasma proteins to the clot [80]. Fibrinogen presents sequences recognized by some integrins; for example, RGD (Arg-Gly-Asp). RGD is one of the most extensively studied. It can bind to multiple integrin species; for example, in platelets, RGD binding integrins, including αIIbβ3, αvβ3, and α5β1, by hydrogen bonds. Besides the sterilization process causing ECM protein denaturation, the functionality of the RGD domain is generally preserved, which minimizes the risk of immune reactivity and increases the number of attached cells when the material is used in a scaffold [81].

Fibrin scaffolds have been used for endothelial cells, leukocytes, platelets, and plasma protein binding [80]. In healthy normal adult joint cartilage, chondrocytes are inactive cells that are considered the structural and functional unit. They exhibit low metabolic and very little renewal activity of the matrix components [82]. They are avascular and thus lack nutrients. In tissue engineering, promoting chondrocyte proliferation and maintaining the chondrocyte phenotype are key points, hence several source cells such as differentiated cells (AC; articular chondrocytes, NC; nasal septum chondrocytes), progenitor cells, multipotent cells (MSC derived from bone marrow, adipose tissue, synovial membrane, synovial fluid, umbilical cord blood, and peripheral blood), or pluripotent cells (ESC; embryonic stem cells and induced pluripotent stem cells) are used [83].

Some advantages of stem cells for cartilage lesion repair are infinite proliferation capacity [84] and excreting chondrocyte-promoting growth factors such as fibroblast growth factor-1(FGF-1) and transforming growth factor-β (TGF-β) [85,86]. Different methods, such as physical stimulation, application of growth factors, and peptides have been proposed to favor chondrocyte proliferation [87]. The latter have been categorized as small molecule compounds that can promote the proliferation of chondrocytes and induce stem cell chondrogenesis. Examples of these compounds include kartogenin, melatonin, simvastatin, dexamethasone, prostaglandin E2, and glucosamine. In terms of cell adhesion, it is necessary to consider the hydrophilicity of the materials. Studies have suggested that the encapsulation of chondrocytes in different biomaterials maintains the cartilage phenotype in vitro for long periods of time. The availability and compatibility of donor tissue has been proposed as the main limitation for this type of procedure [88].

For greater biocompatibility, even proliferation of chondrocytes and osteoblasts, modification of the very nature of the biomaterial has been proposed, as is the case of carbon nanofibers that have been proposed as an alternative in the treatment of bone diseases. Such modifications are at the surface level, thanks to sodium hyaluronate, graphene oxide, silica oxycarbide, or by oxidation.

A greater number of osteoblastic cells and chondrocytes are observed after 72 h into modification scaffolds with of sodium hyaluronate because it promotes the migration, proliferation, and even differentiation of bone cells. Sodium hyaluronate together with hyaluronic acid are incorporated into joint cartilage where they have a biological effect on chondrocytes through CD44 receptors. [89]. Adding well-differentiated allogeneic chondrocytes does not exacerbate the mild host tissue reaction caused by scaffolds, especially those made of porous sponge, probably because the newly synthesized pericellular matrix insulates them from the immune reaction [90].

Studies have shown that scaffolds based on native cartilage ECM components and cartilage specific glycosaminoglycans [91] promote the metabolic activity of chondrocytes and the production of ECM. This could enhance the protection of allogeneic cells against an immune system response.

Embryonic stem cells have been shown to differentiate into chondrocytes in a two-step process, where stem cells initially change their phenotype to chondrogenic progenitors, followed by the differentiation of these progenitor cells into chondrocytes. In vitro differentiation of these stem cells is very effective when combined with a three-dimensional microenvironment, with the addition of growth factors to enhance differentiation [92]. Chondrocyte coculture has also been implemented as a method to support chondrogenesis of MSC and maintain their chondrogenicity [93]. iPSCs are an alternative cell source very similar to ESCs. However, there are certain problems with the clinical application of these cells such as inefficient protocols for their differentiation into functional chondrocytes and the risk of residual undifferentiated cells that may have tumorigenic potential.

As discuss, there are advantages and disadvantages according to the different stages of differentiation (for stem cells) or the use of differentiated cells (such as chondrocytes) in the scaffold in terms of its clinical use. Collection, availability, proliferation, chondrogenic potential, ethical and safety considerations, and the potential for allogeneic or autologous uses are the most important aspects [83]. However, it is important to define the number of cells implanted in the fibrin scaffold, because unlike stem cells, chondrocytes have an accelerated rate of degradation due to their capacity to generate ECM.

Other promising sources of adult stem cells to achieve cartilage regeneration are MSCs; which are not as homogeneous as differentiated cells but can be collected from many tissues (with variations in abundance and chondrogenic potential), thus requiring optimization and adaptation of protocols, as well as a complete characterization. To date, the most favorable results are obtained with bone marrow mesenchymal stem cells (BM-MSC); nevertheless, to enhance the viability, function, and in some cases, achieve the differentiation of the cell seeded into some scaffolds, some growth factors are required.

## 7. Growth Factors on Fibrin Scaffolds

Growth factors are biologically active polypeptides produced by the body that can stimulate cell division, growth, and differentiation. Numerous growth factors work together to regulate cartilage development and homeostasis throughout life. When it comes to cartilage regeneration, the fibrin scaffolds function as a temporary matrix and help prolong the duration of growth factor release and support cell growth and proliferation by providing a temporary matrix that supports tissue remodeling and healing [94].

Most commercial fibrinogen preparations usually contain other plasma proteins such as fibronectin, fibronectin, vitronectin, fibroblast growth factor (FGF), vascular endothelial growth factor (VEGF), insulin-like growth factor-1 (IGF-1), and enzymes, including plasminogen or tissue plasminogen activator; however, the concentrations of these proteins and factors depend on sample collection and preparation methods [95].

Platelets secrete numerous growth factors (e.g., TGF-β, PDGF, VEGF, FGF, HGF, EGF, and IGF) that interact with plasma fibrin, ECM, and tissue cells in a combinatorial, synergistic, and multidirectional manner, offering the opportunity to generate autologous fibrin scaffolds for cartilage repair.

Platelet derivatives can be classified into three different generations based on their characteristics and preparation methods:

- Platelet-rich plasma (PRP) which contains several growth factors implicated in tissue repair such as 100–150 ng/mL of PDGF, 200–400 ng/mL of TGF-b, 4–83 ng/mL of b-FGF, and 115 to 236 ng/mL of IGF [96,97].
- Platelet-rich fibrin (PRF) is prepared from blood samples collected without anticoagulants or biological agents. PRF has been further modified into an advanced form called advanced platelet-rich fibrin (A-PRF), which has a fibrin clot softer than PRF and more platelet cells than PRF [98]. PRF has a solid fibrin matrix that contains a higher concentration of platelets, leucocytes, growth factors (18–148 ng/mL of PDGF, 390–560 ng/mL of TGF-b, around 126.86 ng/mL of b-FGF and around 274 ng/mL of IGF) and adhesive proteins such as fibronectin, fibrinogen, vitronectin, and thrombospondin-1 compared to PRP [99].
- Concentrated growth factors (CGF) (20–175 ng/mL of PDGF, 390–584 ng/mL of TGF-b, around 130.56 ng/mL of b-FGF, 321 pg/mL of IGF, 7.5 pg/mL IL-6) [100]. They can be considered a modified form of PRF. CGF is produced by centrifugation of a blood sample using alternating speed rates. This process leads to a dense fibrin matrix that can promote cell migration, such as fibroblast and endothelial cells [101]. They contain more growth factors than PRP and PRF.

The fibrin scaffold acts as a temporal nesting matrix for cells. It retains and later releases a portion of growth factors delivered “on demand”, which may promote greater biological effects [102], thereby reducing risks of tissue edema and inflammatory responses caused by high concentrations of growth factors [103]. Nevertheless, it has been demonstrated that extremely high growth factor concentrations by PRP are not always beneficial for cell stimulation processes and may not be efficient due to the small number of cell membrane receptors [41]. Along with the short biologically active half-life of some growth factors in PRP, the lack of standardization preparation protocols of PRP in clinical studies may help to explain, at least in part, the variation in PRP therapy results. As a result, it is advised to conduct studies using various PRP injection applications since the growth factors’ short biological half-lives have an impact on biological activity.

There are some discrepancies regarding quantitative amounts of growth factors between CGF and other platelet concentrates. PRP promotes cell proliferation and the synthesis of proteoglycans, hyaluronic acid, and collagen, favoring the lubrication of joint surfaces. TGF-β, PDGF, FGF, and IGF-1 promote cell clearance. TGF-β, PDGF, and IGF regulate ECM synthesis. The functions of VEGF, HGF and FGF-2 include the stimulation of angiogenesis. TGF-β1 is one of the most relevant growth factors in cartilage tissue regeneration as it plays a role in anabolic and regenerative processes, enhancing and modulating anti-inflammatory processes. TGF-β1 is an elementary part of the signaling pathways for cartilage maintenance and is therefore considered important in tissue engineering approaches [104]. However, researchers have reported that CGF gradually delivers growth factors as it degrades at local sites. In joint cartilage, numerous growth factors work together to regulate joint cartilage development and homeostasis throughout life. Fibrin scaffold was shown to augment proliferation, chemotaxis, and chondrocyte viability, induce chondrogenic differentiation and stimulate ECM synthesis via the IGF1R/PI3K/AKT signaling pathway which promotes cartilage regeneration. Additionally, interleukin-1 (IL-1), matrix metalloproteinases (MMP), synthesis of proteoglycans, aggrecan, and type II collage influence chondrogenic differentiation [105].

**Figure 2:**
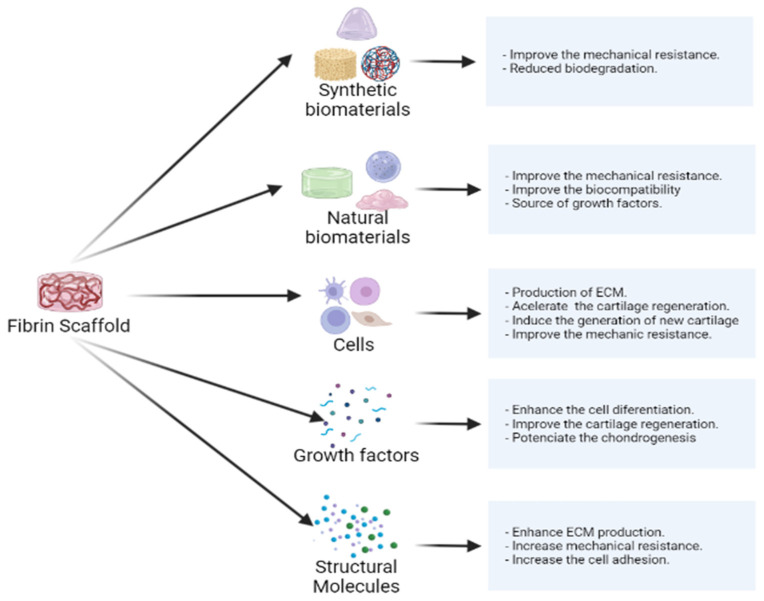
Summary of the advantages of fibrin as a scaffold. Characteristics and components that accompany fibrin scaffolding and promote the repair of chondral tissue.

Fibrin provides ideal natural properties as a scaffold, with growth factors as bioactive components, which are biocompatible as a matrix for cells to migrate. However, to improve its natural properties, some modifications are necessary, as shown in Figure 2.

## 8. Modifications That Promote the Advantages of Fibrin Scaffolds

Fibrin provides an ideal matrix for cells to migrate into; alternatively, it can be further manipulated to enhance its natural properties [49,106,107]. Fibrin modification at a macroscopic scale is achieved by altering the polymerization process, which depends on salt and or thrombin concentrations, pH, and the incorporation of other polymers such as PEG [41,107,108,109].

Hydroxyapatite combined with fibrin stabilizes osteoconductive properties. These properties can lead to successful bone regeneration in maxillofacial and dental surgery. In addition, platelets have been used in this combination to enhance mechanical properties further; moreover, there is evidence that these combinations even improve wound healing [41]. PEG additions to fibrin have been shown to control pore size for encapsulation or cell deposition and migration. One advantage of fibrin-PEG gels is vascularization, as fibrin gives angiogenic properties to the material, and PEG provides interconnected conduits. Compared to other polymers such as PGA or PLLA, the fibrin-PEG system holds distinctive advantages, mainly a potential for high fibrin distribution. This effect has been seen compared to fibrin-PLLA as the gel becomes highly hydrophobic, making fibrin distribution problematic. Other properties include mechanical and adequate pore size control [110].

Hyaluronic acid is a primary component of the ECM and has been studied to develop hydrogels, which are known for their mechanical strength, but when used alone, at high concentrations, to cultivate cells, are rendered unsatisfactory [111], and when combined with fibrin, it is an excellent matrix for cartilage [15,112]. Artificial constructs such as those for developing arteries have been achieved by altering fibrin’s natural properties. Recently, a fibrin-based scaffold using 3D printing technology has been biomanufactured. With the aid of 3D technology, concentric conduits inside natural ECM were engineered to reach highly complex architecture (bi and tri-layered). These remarkable advances permit the development of highly specialized tissue with complex cell deposition [113].

Meanwhile, Brougham et al. recently studied the combined mechanics of a tri-component gel made from fibrin, collagen, and glycosaminoglycans (FCG) a combination tested for its use where dimensional stability is crucial. They showed that this optimized combination of components led to a 6-fold increase in compressibility compared to fibrin alone and a 30-fold increase in tensile strength; furthermore, they successfully incorporated vascular smooth muscle cells, demonstrating high viability and proliferation [114]. While this may seem an odd combination at first, we must consider that many potential combinations can exist, as different combinations have different purposes at hand. The property itself is important when opting for a particular property, whether it be mechanical, hydrophobicity, cell pore control, or any other depending on how the individual components behave in similar or natural conditions [108,115,116] to achieve a functional scaffold that can be biomimetic and biocompatible with the organism.

Although modified fibrin scaffolds contain all the desirable features in vitro to repair a defect, evaluating effective treatments for cartilage injuries in animal models is necessary. However, to choose the best animal model, it is necessary to consider multiple factors. Some of the most relevant are the costs of buying and housing the animals, the type of injury, the dimensions of the defect, the time in which the scaffold is implanted after the lesion, how long progress the will be evaluated, and the number of cells and experimental animals that will be used.

## 9. Applications of Fibrin Scaffolds in Cartilage Engineering

Most of the studies analyzed reported using small animal models (rabbits or minipigs). In two cases, larger animal models such as sheep or horses were used. This situation could be a limitation regarding the evidence found since larger animal models present a greater biomechanical similarity to humans. In three cases, fibrin scaffolds were used in humans, including one randomized clinical trial, which could represent the greatest evidence at the clinical level in this area. Something that all studies had in common, in animals and humans, was that the type of injury treated with fibrin-based implants were mostly complete femoral chondral defects. Another interesting aspect to consider is the follow-up in the studies, where most reported six months or less. For this type of injury, a follow-up of less than one year will hardly provide reliable information regarding the true regenerative capacity of the implant. In patients with a follow-up over 12 months, particularly in clinical studies, lesions were completely filled with evidence of articular cartilage-like repair tissue. Overall, the results of the in vivo studies are encouraging, indicating that fibrin-based implants are an option in the treatment of cartilage injuries using tissue engineering, especially when the fibrin matrix fibrin is combined with cells or some other biomaterial. The full analysis and main outcomes from these studies are depicted in Table 4.

## 10. Conclusions

Many scaffolds offer tissue engineering as an alternative for degenerative joint disease. Stem cells and autologous chondrocytes are the gold standards for joint injuries; however, due to the nature of this cartilage tissue, it is difficult to maintain viability and the stage of differentiation once these are implanted. Other approaches have been sought that allow better integration and promote chondrocyte differentiation. Fibrin has been shown to have ideal characteristics useful in joint cartilage injuries. It is easy to obtain at a low cost, is biodegradable and biocompatible, and could also provide growth factors that can improve cartilage regeneration; however, these properties can be improved with some modifications, such as mixture with biological or synthetic biomaterials and chemical modification. These modifications give it greater hardness, durability, anchorage for cells, or even functionality as a delivery system of growth factors, among other aspects relevant to validating them through in vitro and in vivo assays.

The even greater challenge is to establish a consensus on the main parameters to evaluate during the construction of the scaffold and in vivo assays, as an animal model, type of injury, number of cells seeded, implant methods, and other characteristics that are important to promote cartilage injury repair. We hope that in the future, the deep characterization of the scaffold can provide more standardized protocols that allow chondrogenic potential to be better assessed.

## Figures and Tables

**Table 3:** Examples of fibrin scaffolds or fibrin mixed with other biomaterial for in vivo applications.

Scaffold	Animal Model	Specific Sites of Implantation	Time of Evaluation	Results	Ref.
Fibrin scaffold	Male rats	Subcutaneously transplanted in skin (ectopic)	14 days	After 14 days of in vitro chondrogenic differentiation of human adipose derived stem cells, the fibrin scaffold, in which the cells were differentiated, were implanted. In vivo differentiation of cells under the skin increased the amount of cartilage matrix constituents such as proteoglycans.	[69]
Polyglycolic acid (PGA)-fibrin scaffolds	Immuno-compromised athymic mice	Subcutaneously transplanted in skin (ectopic)	4 weeks	Subcutaneous transplantation of human infant hip chondrocytes loaded in PGA-fibrin scaffolds, added with human platelet-rich plasma, in nude mice, showed the formation of hyaline-like cartilage, rich in type II and type X collagen.	[70]
Fibrin hydrogel	Immune deficient SCID mice	Subcutaneously transplanted in back skin (ectopic)	4 weeks	Pro-chondrogenic and pro-hypertrophic bioactive fibrin hydrogel with high potential to promote the integration of cartilage scaffolds with bone. Fibrin hydrogel promises to overcome poor fixation of biomaterials in cartilage defects facilitating their long-term regeneration.	[71]
Fibrin gel	5–6 months age New Zealand white rabbit	Implantation in injured knee joint	6 weeks	Fibrocartilaginous repair tissues, containing the hyaline cartilage marker collagen type 2.	[72]
Polyurethane fibrin composite	Female adult New Zealand White Rabbits	Injured auricular cartilage	4 and 12 weeks	After 12 weeks in vivo, there was a production of cartilage extracellular matrix components. Also, there is gene expression of specific marker genes for mature cartilage, such as SOX-9 and collagen II.	[73]

**Table 4:** Examples of in vivo studies of fibrin scaffold for regeneration or repair of chondral lesions.

Study Model	Implant Used	Follow-Up	Results	Ref.
Sheep Full-thickness chondral defects on femoral condyle	Autologous fibrin scaffold	12 weeks	Good integration with surrounding cartilage. Nearly normal appearance. Cells resembled chondrocytes embedded within cartilaginous-like matrix. Histological section revealed accumulated proteoglycans.	[117]
Case series (human) Deep cartilage defects on femoral condyle	Fibrin gel/autologous chondrocytes	24 months (clinical) 12 months (second look arthroscopy/histological)	Most patients had excellent/good results. Clinical scores improved. Synthesis of GAG and type II collagen in implants. Grafted areas with good filling; some grafts with fibrillations or mild hypertrophy; most tissue repair well integrated.	[118]
Minipigs Full-thickness chondral defects on femoral condyle	Fibrin matrix/acellular cartilage matrix/autologous chondrocytes	12 weeks	Surface of the repaired joint cartilage (fibrin, cartilage matrix, and chondrocytes) was porcelain white, slightly transparent, and smooth but thinner than normal cartilage. Comparable tissue thickness in the repaired region with the surrounding tissue; few fibrous connective tissues distributed on the boundary. Complete and homogeneous distribution of GAGs and type II collagen, less than normal cartilage.	[119]
Minipigs Full-thickness chondral defects on femoral trochlea	Commercial fibrin matrix/commercial hyaluronic acid/autologous chondrocytes	24 weeks	Type II collagen revealed positivity in the newly formed cartilage on the borders of the defects. Type II collagen was less present in the center of the defect. Biomechanical properties of fibrin/HA composite hydrogel at 6 months comparable with native cartilage. Presence of a noncellular transient zone followed by a layer of isogenous groups of chondrocytes merged with fibrocartilaginous tissue at the center.	[112]
Adult horses Full-thickness chondral defects on femoral condyle	Autologous platelet-enriched fibrin/bone-marrow-derived mesenchymal stem cells	12 months	The addition of BMDMSCs to APEF did not enhance cartilage repair and stimulated bone formation in some cartilage defects. The middle-to-superficial part of the repair had a more fibrous, hypocellular appearance with an absence of GAG staining.	[120]
Rabbit Osteochondral defects on femoral trochlear groove	Autologous platelet-rich plasma gel/allogenic chondrocytes	12 and 36 weeks	Similarity of repaired tissue with normal cartilage. Relatively complete integration with surrounding cartilage. Defects were mainly filled by fibrocartilaginous tissue. Lateral and basal integration was relatively suitable. Presence of type II collagen and proteoglycans less than normal cartilage.	[121]
Randomized clinical trial Symptomatic cartilage lesion on the femoral condyle	Commercial fibrin scaffold/adipose-derived stem cells	24 months	Significantly more patients with fibrin/stem cells (80%) exhibited normal or nearly normal repair tissue signal intensity (MRI findings). Intermediate degree of staining for safranin O (proteoglycan) and type II collagen.	[122]
Minipigs Osteochondral defects on femoral condyle	Autologous platelet-rich fibrin/autologous cartilage fragments (0.25 cm^3^) 12 months	6 months	Healing almost complete, reparative tissue appeared to be well integrated at the margins of the repair site, flush and smooth surfaces were observed on the repaired cartilage. Better stiffness compared with controls. Relatively smooth repaired hyaline-like cartilage containing columnar arrangements of chondrocytes. The regenerated tissues appeared to be integrated with the normal hyaline cartilage as well as with the underlying subchondral bone.	[123]
Rabbits Full-thickness osteochondral defects	Commercial fibrin matrix/allogenic chondrocytes or autologous bone-marrow-derived mesenchymal stem cells	12 weeks	Regenerated tissue showed a mixture of hyaline cartilage and fibrocartilage, well connected to the surrounding normal cartilage. Higher expression of type II collagen, clearer configuration and distribution of chondrocytes and collagen; higher concentrations of GAG regarding controls.	[124]
Rabbits Full-thickness osteochondral defects on trochlear groove	Commercial fibrin gel/autologous redifferentiated chondrocytes	6 weeks	Repair tissues from dedifferentiated cell implants resembled fibrocartilage. They contained both Col 1 and Col 2 as well as ACAN. Average ratio of Col 2:Col 1 was greater for tissues formed by dedifferentiated cells than for tissues formed by redifferentiated cells. Redifferentiation of passaged chondrocytes does not improve defect repair in the first 6 weeks.	[72]
Rabbits Full-thickness osteochondral defects on trochlear groove	Autologous fibrin glue/menstrual blood-derived stem cells	12–24 weeks	Defects were filled with hyaline cartilage-like tissue with proper integration, high content of glycosaminoglycan, and the existence of collagen fibers, especially collagen type II.	[125]
Case series (human) Full thickness cartilage lesion in medial femoral condyle	Commercial fibrinogen and thrombin/autologous chondrocytes	12 months	Arthroscopic evaluation indicated that cartilage repair was adequate (mild hypertrophy existed). Histological analysis indicated high deposition of GAGs, adequate type II collagen expression, and higher type II collagen expression over type I collagen.	[126]
Minipigs Full thickness cartilage defects in medial femoral condyle	Platelet-rich fibrin/diced cartilage autografts	6 months	Most of the repair tissue stained positively for Col II but negatively for Col I. Repair tissue integrated with contiguous native tissue and the subchondral bone. Repair tissue integrated with contiguous native tissue and the subchondral bone.	[127]
Rabbits Full thickness cartilage defects in femoral trochlear groove	Platelet-rich fibrin membrane alone	24 weeks	Repaired cartilage covered the defect well, both edges of the repaired cartilage well integrated, smooth surface. Defect was not filled with chondrocyte-like cells and cartilage matrix. Margins of the repaired cartilage were well integrated. Type II collagen staining in the area of repair was observed.	[128]
Rabbits Full thickness cartilage defects in femoral trochlear groove	Commercial xenogeneic porcine fibrin sealant/autologous chondrocytes	6 months	GAG content and type II collagen expression were consistent with the surrounding normal cartilage, and the integration of the new tissue was continuous and smooth. best reparative effect in the fibrin matrix plus autologous chondrocytes. Better mechanical properties than fibrin matrix alone.	[129]

## Data Availability

Not applicable.
